# 4-Benzyl-6-bromo-2-phenyl-4*H*-imidazo[4,5-*b*]pyridine

**DOI:** 10.1107/S160053681001038X

**Published:** 2010-03-27

**Authors:** Y. Ouzidan, S. Obbade, F. Capet, El Mokhtar Essassi, Seik Weng Ng

**Affiliations:** a Laboratoire de Chimie Organique Appliquée, Faculté des Sciences et Techniques, Université Sidi Mohamed Ben Abdallah, Fés, Morocco; b Laboratoire d’Electrochimie et de Physicochimie des Matériaux et des Interfaces, Domaine Universitaire, 38402 St Martin d’Hères Cedex, Grenoble, France; c Unité de Catalyse et de Chimie du Solide, Ecole Nationale Supérieure de Chimie de Lille, Lille, France; d Laboratoire de Chimie Organique Hétérocyclique, Pôle de Compétences Pharmacochimie, Université Mohammed V-Agdal, BP 1014 Avenue Ibn Batout, Rabat, Morocco; e Department of Chemistry, University of Malaya, 50603 Kuala Lumpur, Malaysia

## Abstract

The imidazopyridine fused ring in the title compound, C_19_H_14_BrN_3_, is almost coplanar with the phenyl ring at the 2-position of the five-membered ring [dihedral angle = 2.4 (1). The crystal structure features short Br⋯Br contacts [3.562 (1) Å].

## Related literature

For the synthesis of imidazo[4,5-*b*]pyridines, see: Aridoss *et al.* (2006 ▶); Benham *et al.* (1995 ▶); Cundy *et al.* (1997 ▶); Kale *et al.* (2009 ▶); Walsh *et al.* (1994 ▶); Zaki & Proença (2007 ▶).
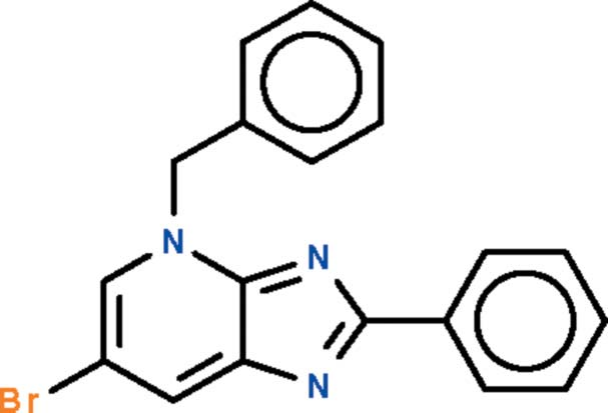


## Experimental

### 

#### Crystal data

C_19_H_14_BrN_3_
*M* *_r_* = 364.24
Monoclinic, 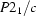

*a* = 8.6613 (6) Å
*b* = 19.7631 (13) Å
*c* = 9.3683 (6) Å
β = 99.647 (3)°
*V* = 1580.93 (18) Å^3^
*Z* = 4
Mo *K*α radiation
μ = 2.60 mm^−1^
*T* = 293 K
0.28 × 0.24 × 0.20 mm

#### Data collection

Bruker X8 APEXII diffractometer
Absorption correction: multi-scan (*SADABS*; Sheldrick, 1996 ▶) *T*_min_ = 0.529, *T*_max_ = 0.624
57936 measured reflections
4613 independent reflections
3492 reflections with *I* > 2σ(*I*)
*R*_int_ = 0.035

#### Refinement

*R*[*F* ^2^ > 2σ(*F* ^2^)] = 0.032
*wR*(*F* ^2^) = 0.098
*S* = 1.00
4613 reflections
208 parameters
H-atom parameters constrained
Δρ_max_ = 0.63 e Å^−3^
Δρ_min_ = −0.51 e Å^−3^

### 

Data collection: *APEX2* (Bruker, 2008 ▶); cell refinement: *SAINT* (Bruker, 2008 ▶); data reduction: *SAINT*; program(s) used to solve structure: *SHELXS97* (Sheldrick, 2008 ▶); program(s) used to refine structure: *SHELXL97* (Sheldrick, 2008 ▶); molecular graphics: *X-SEED* (Barbour, 2001 ▶); software used to prepare material for publication: *publCIF* (Westrip, 2010 ▶).

## Supplementary Material

Crystal structure: contains datablocks global, I. DOI: 10.1107/S160053681001038X/pk2232sup1.cif

Structure factors: contains datablocks I. DOI: 10.1107/S160053681001038X/pk2232Isup2.hkl

Additional supplementary materials:  crystallographic information; 3D view; checkCIF report

## Figures and Tables

**Fig. 1.**
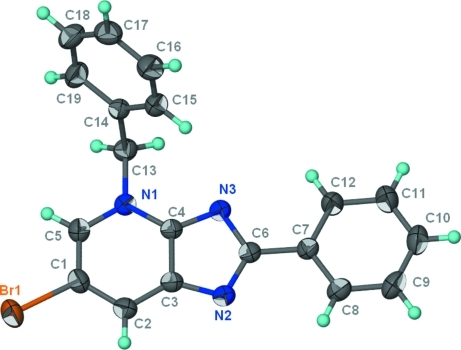
Thermal ellipsoid plot (Barbour, 2001) of C19H14BrN3 at the 50% probability level. Hydrogen atoms are drawn as spheres of arbitrary radius.

## supplementary crystallographic information

### Comment

Imidazo[4,5-*b*]pyridines are a class of sedative drugs exemplified by *Zolpidem*, *Alpidem*, *Saripidem* and *Necopidem*. There is intense interest in designing new synthetic routes; for example, an eco-friendly synthesis by oxidation in aqueous medium has been claimed (Kale *et al.*, 2009). Other methods require more than one step (Aridoss *et al.*, 2006; Benham *et al.*, 1995; Cundy *et al.*, 1997; Walsh *et al.*, 1994; Zaki & Proença, 2007).

We have been able to react 6-bromo-2-phenyl-1*H*-imidazo[4,5-*b*]pyridine with benzyl chloride in the presence of a catalytic quantity of tetra-*n*-butylammonium bromide under mild conditions to furnish the title compound (Scheme I, Fig. 1). The imidazopyridine fused-ring in C_19_H_14_BrN_3_ is co-planar with the phenyl ring at the 2-position [dihedral angle 2.4 (1) °]. In the five-membered imidazo portion, the carbon–nitrogen bond whose carbon atom is also connected to the pyridine nitrogen atom is predominantly a double bond [1.329 (2) Å], whereas the carbon–nitrogen bond whose atom is connected to the pyridine carbon atom is predominantly a single bond [1.372 (2) Å].

### Experimental

To a solution of the 6-bromo-2-phenyl-1*H*-imidazo[4,5-*b*]pyridine (0.30 g, 1.09 mmol), potassium carbonate (0.20 g, 1.42 mmol) and tetra-*n*-butylammonium bromide (0.04 g (0,1 mmol) in DMF (15 ml) was added benzyl chloride (0.15 ml, 1.31 mmol). Stirring was continued at room temperature for 12 hours. The salt was removed by filtration and the filtrate concentrated under reduced pressure. The residue was separated by chromatography on a column of silica gel with ethyl acetate/hexane (1/1) as eluent. Brown crystals were isolated when the solvent was allowed to evaporate.

### Refinement

Carbon-bound H-atoms were placed in calculated positions (C—H 0.93–0.97 Å) and were included in the refinement in the riding model approximation, with *U*(H) set to 1.2*U*(C).

### Crystal data

**Table:**

C_19_H_14_BrN_3_	*F*(000) = 736
*M**_r_* = 364.24	*D*_x_ = 1.530 Mg m^−^^3^
Monoclinic, *P*2_1_/*c*	Mo *K*α radiation, λ = 0.71073 Å
Hall symbol: -P 2ybc	Cell parameters from 9876 reflections
*a* = 8.6613 (6) Å	θ = 2.4–27.2°
*b* = 19.7631 (13) Å	µ = 2.60 mm^−^^1^
*c* = 9.3683 (6) Å	*T* = 293 K
β = 99.647 (3)°	Prism, brown
*V* = 1580.93 (18) Å^3^	0.28 × 0.24 × 0.20 mm
*Z* = 4	

### Data collection

**Table:**

Bruker X8 APEXII diffractometer	4613 independent reflections
Radiation source: fine-focus sealed tube	3492 reflections with *I* > 2σ(*I*)
graphite	*R*_int_ = 0.035
φ and ω scans	θ_max_ = 30.1°, θ_min_ = 2.4°
Absorption correction: multi-scan (*SADABS*; Sheldrick, 1996)	*h* = −12→11
*T*_min_ = 0.529, *T*_max_ = 0.624	*k* = −27→27
57936 measured reflections	*l* = −13→13

### Refinement

**Table:**

Refinement on *F*^2^	Primary atom site location: structure-invariant direct methods
Least-squares matrix: full	Secondary atom site location: difference Fourier map
*R*[*F*^2^ > 2σ(*F*^2^)] = 0.032	Hydrogen site location: inferred from neighbouring sites
*wR*(*F*^2^) = 0.098	H-atom parameters constrained
*S* = 1.00	*w* = 1/[σ^2^(*F*_o_^2^) + (0.0518*P*)^2^ + 0.5269*P*] where *P* = (*F*_o_^2^ + 2*F*_c_^2^)/3
4613 reflections	(Δ/σ)_max_ = 0.001
208 parameters	Δρ_max_ = 0.63 e Å^−^^3^
0 restraints	Δρ_min_ = −0.50 e Å^−^^3^

### Fractional atomic coordinates and isotropic or equivalent isotropic displacement parameters (Å^2^)

**Table:**

	*x*	*y*	*z*	*U*_iso_*/*U*_eq_	
Br1	0.10158 (3)	0.475089 (12)	0.85824 (2)	0.06483 (10)	
N1	0.29485 (16)	0.43405 (7)	0.49901 (15)	0.0385 (3)	
N2	0.26154 (17)	0.60722 (7)	0.40338 (16)	0.0424 (3)	
N3	0.35446 (17)	0.50867 (7)	0.31224 (15)	0.0387 (3)	
C1	0.1792 (2)	0.48667 (9)	0.68312 (19)	0.0454 (4)	
C2	0.1807 (2)	0.55060 (9)	0.62007 (19)	0.0458 (4)	
H2	0.1429	0.5886	0.6616	0.055*	
C3	0.2408 (2)	0.55460 (8)	0.49376 (18)	0.0392 (3)	
C4	0.29944 (19)	0.49489 (8)	0.43321 (17)	0.0367 (3)	
C5	0.2352 (2)	0.43003 (9)	0.62362 (18)	0.0437 (4)	
H5	0.2322	0.3884	0.6694	0.052*	
C6	0.32810 (19)	0.57716 (8)	0.30027 (17)	0.0379 (3)	
C7	0.37246 (19)	0.61435 (8)	0.17772 (18)	0.0390 (3)	
C8	0.3423 (2)	0.68335 (9)	0.1595 (2)	0.0459 (4)	
H8	0.2938	0.7067	0.2261	0.055*	
C9	0.3845 (2)	0.71728 (10)	0.0425 (2)	0.0547 (5)	
H9	0.3644	0.7634	0.0310	0.066*	
C10	0.4557 (3)	0.68322 (11)	−0.0569 (2)	0.0574 (5)	
H10	0.4829	0.7062	−0.1357	0.069*	
C11	0.4869 (3)	0.61517 (11)	−0.0399 (2)	0.0624 (5)	
H11	0.5353	0.5922	−0.1069	0.075*	
C12	0.4459 (3)	0.58097 (10)	0.0773 (2)	0.0538 (5)	
H12	0.4679	0.5350	0.0888	0.065*	
C13	0.3544 (2)	0.37271 (8)	0.43491 (19)	0.0427 (3)	
H13A	0.4041	0.3433	0.5121	0.051*	
H13B	0.4331	0.3859	0.3778	0.051*	
C14	0.22599 (19)	0.33413 (8)	0.34017 (17)	0.0380 (3)	
C15	0.1392 (2)	0.36407 (9)	0.21824 (19)	0.0481 (4)	
H15	0.1605	0.4084	0.1945	0.058*	
C16	0.0214 (3)	0.32854 (11)	0.1321 (2)	0.0574 (5)	
H16	−0.0379	0.3494	0.0522	0.069*	
C17	−0.0082 (3)	0.26217 (11)	0.1643 (2)	0.0585 (5)	
H17	−0.0859	0.2380	0.1050	0.070*	
C18	0.0769 (3)	0.23206 (10)	0.2833 (3)	0.0598 (5)	
H18	0.0570	0.1873	0.3047	0.072*	
C19	0.1930 (2)	0.26789 (9)	0.3728 (2)	0.0517 (4)	
H19	0.2487	0.2473	0.4548	0.062*	

### Atomic displacement parameters (Å^2^)

**Table:**

	*U*^11^	*U*^22^	*U*^33^	*U*^12^	*U*^13^	*U*^23^
Br1	0.08695 (19)	0.06436 (15)	0.05128 (13)	0.00593 (10)	0.03507 (11)	0.00630 (9)
N1	0.0419 (7)	0.0355 (6)	0.0382 (7)	0.0001 (5)	0.0074 (5)	0.0003 (5)
N2	0.0515 (8)	0.0338 (6)	0.0437 (7)	−0.0007 (6)	0.0131 (6)	−0.0028 (5)
N3	0.0443 (7)	0.0337 (6)	0.0394 (7)	−0.0009 (5)	0.0104 (5)	−0.0012 (5)
C1	0.0501 (10)	0.0498 (10)	0.0386 (8)	−0.0024 (7)	0.0140 (7)	0.0004 (7)
C2	0.0535 (10)	0.0417 (9)	0.0444 (9)	−0.0007 (7)	0.0147 (7)	−0.0056 (7)
C3	0.0431 (8)	0.0349 (7)	0.0402 (8)	−0.0015 (6)	0.0088 (6)	−0.0046 (6)
C4	0.0390 (8)	0.0345 (7)	0.0366 (7)	−0.0020 (6)	0.0061 (6)	−0.0035 (6)
C5	0.0485 (9)	0.0430 (8)	0.0403 (8)	−0.0019 (7)	0.0094 (7)	0.0036 (7)
C6	0.0407 (8)	0.0345 (7)	0.0385 (7)	−0.0028 (6)	0.0068 (6)	−0.0023 (6)
C7	0.0420 (8)	0.0353 (7)	0.0395 (7)	−0.0036 (6)	0.0064 (6)	−0.0004 (6)
C8	0.0471 (9)	0.0360 (8)	0.0553 (10)	−0.0020 (7)	0.0105 (8)	−0.0007 (7)
C9	0.0567 (11)	0.0391 (9)	0.0678 (12)	−0.0031 (8)	0.0092 (9)	0.0118 (8)
C10	0.0647 (12)	0.0560 (11)	0.0530 (10)	−0.0070 (9)	0.0141 (9)	0.0153 (9)
C11	0.0851 (15)	0.0567 (12)	0.0516 (10)	0.0037 (11)	0.0298 (10)	0.0062 (9)
C12	0.0782 (13)	0.0393 (9)	0.0486 (10)	0.0062 (9)	0.0240 (9)	0.0040 (7)
C13	0.0430 (9)	0.0371 (8)	0.0483 (9)	0.0064 (7)	0.0091 (7)	0.0015 (7)
C14	0.0419 (8)	0.0331 (7)	0.0416 (8)	0.0029 (6)	0.0147 (6)	−0.0002 (6)
C15	0.0608 (11)	0.0390 (8)	0.0446 (9)	−0.0013 (8)	0.0092 (8)	0.0021 (7)
C16	0.0660 (12)	0.0574 (11)	0.0462 (10)	−0.0037 (9)	0.0015 (9)	−0.0030 (8)
C17	0.0603 (12)	0.0584 (12)	0.0587 (11)	−0.0140 (9)	0.0152 (9)	−0.0156 (9)
C18	0.0683 (13)	0.0396 (9)	0.0754 (14)	−0.0103 (9)	0.0233 (11)	−0.0022 (9)
C19	0.0583 (11)	0.0388 (9)	0.0593 (11)	0.0013 (8)	0.0139 (9)	0.0088 (8)

### Geometric parameters (Å, °)

**Table:**

Br1—C1	1.8882 (18)	C9—H9	0.9300
N1—C4	1.355 (2)	C10—C11	1.376 (3)
N1—C5	1.356 (2)	C10—H10	0.9300
N1—C13	1.483 (2)	C11—C12	1.385 (3)
N2—C6	1.344 (2)	C11—H11	0.9300
N2—C3	1.372 (2)	C12—H12	0.9300
N3—C4	1.329 (2)	C13—C14	1.508 (2)
N3—C6	1.374 (2)	C13—H13A	0.9700
C1—C5	1.375 (3)	C13—H13B	0.9700
C1—C2	1.396 (3)	C14—C19	1.385 (2)
C2—C3	1.373 (2)	C14—C15	1.390 (2)
C2—H2	0.9300	C15—C16	1.382 (3)
C3—C4	1.438 (2)	C15—H15	0.9300
C5—H5	0.9300	C16—C17	1.379 (3)
C6—C7	1.468 (2)	C16—H16	0.9300
C7—C12	1.388 (3)	C17—C18	1.366 (3)
C7—C8	1.394 (2)	C17—H17	0.9300
C8—C9	1.385 (3)	C18—C19	1.390 (3)
C8—H8	0.9300	C18—H18	0.9300
C9—C10	1.376 (3)	C19—H19	0.9300
			
C4—N1—C5	119.22 (14)	C11—C10—H10	120.0
C4—N1—C13	120.17 (14)	C9—C10—H10	120.0
C5—N1—C13	120.61 (14)	C10—C11—C12	119.8 (2)
C6—N2—C3	102.99 (13)	C10—C11—H11	120.1
C4—N3—C6	101.13 (13)	C12—C11—H11	120.1
C5—C1—C2	122.44 (16)	C11—C12—C7	120.86 (18)
C5—C1—Br1	117.06 (13)	C11—C12—H12	119.6
C2—C1—Br1	120.49 (14)	C7—C12—H12	119.6
C3—C2—C1	116.66 (16)	N1—C13—C14	112.30 (13)
C3—C2—H2	121.7	N1—C13—H13A	109.1
C1—C2—H2	121.7	C14—C13—H13A	109.1
N2—C3—C2	133.11 (16)	N1—C13—H13B	109.1
N2—C3—C4	106.70 (14)	C14—C13—H13B	109.1
C2—C3—C4	120.18 (16)	H13A—C13—H13B	107.9
N3—C4—N1	127.72 (15)	C19—C14—C15	118.70 (17)
N3—C4—C3	111.64 (14)	C19—C14—C13	120.47 (16)
N1—C4—C3	120.64 (15)	C15—C14—C13	120.82 (15)
N1—C5—C1	120.85 (16)	C16—C15—C14	120.56 (17)
N1—C5—H5	119.6	C16—C15—H15	119.7
C1—C5—H5	119.6	C14—C15—H15	119.7
N2—C6—N3	117.54 (14)	C17—C16—C15	120.1 (2)
N2—C6—C7	122.76 (14)	C17—C16—H16	119.9
N3—C6—C7	119.70 (14)	C15—C16—H16	119.9
C12—C7—C8	118.69 (17)	C18—C17—C16	119.88 (19)
C12—C7—C6	120.15 (15)	C18—C17—H17	120.1
C8—C7—C6	121.17 (16)	C16—C17—H17	120.1
C9—C8—C7	120.11 (18)	C17—C18—C19	120.47 (18)
C9—C8—H8	119.9	C17—C18—H18	119.8
C7—C8—H8	119.9	C19—C18—H18	119.8
C10—C9—C8	120.42 (18)	C14—C19—C18	120.25 (18)
C10—C9—H9	119.8	C14—C19—H19	119.9
C8—C9—H9	119.8	C18—C19—H19	119.9
C11—C10—C9	120.08 (18)		
			
C5—C1—C2—C3	0.3 (3)	N2—C6—C7—C12	177.72 (18)
Br1—C1—C2—C3	179.25 (13)	N3—C6—C7—C12	−2.4 (2)
C6—N2—C3—C2	179.20 (19)	N2—C6—C7—C8	−2.2 (3)
C6—N2—C3—C4	−0.16 (17)	N3—C6—C7—C8	177.69 (16)
C1—C2—C3—N2	−179.77 (18)	C12—C7—C8—C9	0.4 (3)
C1—C2—C3—C4	−0.5 (3)	C6—C7—C8—C9	−179.61 (16)
C6—N3—C4—N1	−179.97 (16)	C7—C8—C9—C10	0.2 (3)
C6—N3—C4—C3	−0.15 (18)	C8—C9—C10—C11	−0.6 (3)
C5—N1—C4—N3	179.35 (16)	C9—C10—C11—C12	0.2 (4)
C13—N1—C4—N3	−0.7 (3)	C10—C11—C12—C7	0.5 (4)
C5—N1—C4—C3	−0.5 (2)	C8—C7—C12—C11	−0.8 (3)
C13—N1—C4—C3	179.47 (15)	C6—C7—C12—C11	179.25 (19)
N2—C3—C4—N3	0.21 (19)	C4—N1—C13—C14	−94.67 (18)
C2—C3—C4—N3	−179.25 (16)	C5—N1—C13—C14	85.26 (19)
N2—C3—C4—N1	−179.95 (15)	N1—C13—C14—C19	−119.47 (17)
C2—C3—C4—N1	0.6 (2)	N1—C13—C14—C15	60.9 (2)
C4—N1—C5—C1	0.3 (2)	C19—C14—C15—C16	0.5 (3)
C13—N1—C5—C1	−179.68 (16)	C13—C14—C15—C16	−179.83 (17)
C2—C1—C5—N1	−0.2 (3)	C14—C15—C16—C17	−1.8 (3)
Br1—C1—C5—N1	−179.17 (13)	C15—C16—C17—C18	1.4 (3)
C3—N2—C6—N3	0.1 (2)	C16—C17—C18—C19	0.2 (3)
C3—N2—C6—C7	180.00 (15)	C15—C14—C19—C18	1.1 (3)
C4—N3—C6—N2	0.05 (19)	C13—C14—C19—C18	−178.56 (17)
C4—N3—C6—C7	−179.88 (14)	C17—C18—C19—C14	−1.5 (3)

## References

[bb1] Aridoss, G., Balasubramanian, S., Parthiban, P. & Kabilan, S. (2006). *Eur. J. Med. Chem. ***41**, 268–275. 10.1016/j.ejmech.2005.10.014 16380194

[bb2] Barbour, L. J. (2001). *J. Supramol. Chem.* **1**, 189–191.

[bb3] Benham, C. D., Blackburn, T. P., Johns, A., Kotecha, N. R., Martin, R. T., Thomas, D. R., Thompson, M. & Ward, R. W. (1995). *Bioorg. Med. Chem. Lett.* **5**, 2455–2460.

[bb4] Bruker (2008). *APEX2* and *SAINT* Bruker AXS Inc., Madison, Wisconsin, USA.

[bb5] Cundy, D. J., Holan, G., Otaegui, M. & Simpson, G. W. (1997). *Bioorg. Med. Chem. Lett.* **7**, 669–674.

[bb6] Kale, R. P., Shaikh, M. U., Jadhav, G. R. & Gill, C. H. (2009). *Tetrahedron Lett.* **50**, 1780–1782.

[bb7] Sheldrick, G. M. (1996). *SADABS* University of Göttingen, Germany.

[bb8] Sheldrick, G. M. (2008). *Acta Cryst.* A**64**, 112–122. 10.1107/S0108767307043930 18156677

[bb9] Walsh, T. F., Fitch, K. J., MacCoss, M., Chang, R. S. L., Kivlighn, S. D., Lotti, V. J., Siegl, P. K. S., Patchett, A. & Greenlee, W. J. (1994). *Bioorg. Med. Chem. Lett. ***4**, 219–222.

[bb10] Westrip, S. P. (2010). *publCIF* In preparation.

[bb11] Zaki, M. E. A. & Proença, M. F. (2007). *Tetrahedron*, **63**, 3745–3753.

